# The Role of Genetic Variants in the Susceptibility of Noise-Induced Hearing Loss

**DOI:** 10.3389/fncel.2022.946206

**Published:** 2022-07-12

**Authors:** Xue-min Chen, Xin-miao Xue, Ning Yu, Wei-wei Guo, Shuo-long Yuan, Qing-qing Jiang, Shi-ming Yang

**Affiliations:** ^1^Medical School of Chinese PLA, Beijing, China; ^2^Senior Department of Otolaryngology-Head & Neck Surgery, Chinese PLA General Hospital, Beijing, China; ^3^National Clinical Research Center for Otolaryngologic Diseases, Beijing, China; ^4^State Key Lab of Hearing Science, Ministry of Education, Beijing, China; ^5^Beijing Key Lab of Hearing Impairment Prevention and Treatment, Beijing, China

**Keywords:** genes, noised-induced hearing loss, noise prevention, susceptibility, genetic variants

## Abstract

Noised-induced hearing loss (NIHL) is an acquired, progressive neurological damage caused by exposure to intense noise in various environments including industrial, military and entertaining settings. The prevalence of NIHL is much higher than other occupational injuries in industrialized countries. Recent studies have revealed that genetic factors, together with environmental conditions, also contribute to NIHL. A group of genes which are linked to the susceptibility of NIHL had been uncovered, involving the progression of oxidative stress, potassium ion cycling, cilia structure, heat shock protein 70 (HSP70), DNA damage repair, apoptosis, and some other genes. In this review, we briefly summarized the studies primary in population and some animal researches concerning the susceptible genes of NIHL, intending to give insights into the further exploration of NIHL prevention and individual treatment.

## Introduction

The auditory system helps people to hear sound, understand language, and even distinguish people or objects by recognizing different sounds. Any organic or functional impairment of the auditory pathway can lead to hearing impairment. According to a WHO report (Castaneda et al., 2019), more than 100 million people in East Asia are at risk of disabling hearing loss, leading to lifelong disability, and deafness has become one of the major problems affecting their life quality. Sensorineural hearing loss may be caused by pathological changes in the Corti’s organ of the inner ear, the auditory nerve, or the auditory cortex. It is characterized by the impairment of sound perceptive and analytic ability, and classified as drug-induced hearing loss, presbycusis, hereditary hearing loss, noise-induced hearing loss (NIHL) and others. Although cochlear implant technology has been increasingly advanced in the treatment of hearing loss, its therapeutic effect varies with different lesion sites, therefore, sensorineural hearing loss remains one of the most challenging medical problems.

Noise pollution is one of the seven public hazards in modern society. NIHL, one of the hot spots of social concern, is the second cause of hearing loss in adults, and more than 6% of the global population is affected by NIHL according to WHO data (Sliwinska-Kowalska and Zaborowski, 2017). NIHL is an acquired hearing loss caused by long-term exposure of the auditory system to noise generated by construction, entertainment, industrial production, military equipment or others, and its incidence is only behind presbycusis among all the types of sensorineural hearing loss (Miao et al., 2019). The A-frequency weighting network (dBA) is normally utilized to measure the levels of noise in decibels (dB) of sound pressure, indicating the risk of NIHL (Varela-Nieto et al., 2020). Moreover, the principal requirements for the diagnosis of NIHL are high-frequency hearing impairment, jeopardous amount of noise exposure and recognizable high-frequency audiometric notch or bulge (Coles et al., 2000). Addition to the auditory symptoms such as hearing descending, hearing allergy, tinnitus, the noise damage may also present as mental disorder, digestive disorder or some other organic dysfunction (Skogstad et al., 2016; Hahad et al., 2019).

Long-term noise exposure can lead to damage of peripheral auditory system, including the structure of cochlea hair cells, cilia, supporting cells, and tectorial membrane (Wang et al., 2002), hitting the external layer of hair cells the most, and the Corti’s organ and spiral ganglion may also undergo degenerative changes (Henderson et al., 2006). The main manifestations of which are increased hearing threshold, decreased auditory sensitivity and speech resolution, tinnitus, and auditory hypersensitivity. A “V”-shaped depression appears at 4k Hz on the audiogram, which is called “V”-shaped notch hearing loss (Carroll et al., 2017). Low intensity or short time noise exposure can cause temporary changes of the auditory nerve synaptic transmitter, resulting in temporary hearing loss which could return to normal after the noise ceased, in terms of temporary threshold shift (TTS) (Kurabi et al., 2017). High intensity or long-time noise exposure causes damages on both hair cells and auditory nerve, resulting in hearing loss that could not be restored, which is called permanent threshold shift (PTS), and eventually leads to sensorineural hearing loss (Liberman, 2016).

Long-term noise exposure may also cause damage to the central auditory system, which mainly occurred in the cochlear nucleus, olivary nucleus, medial geniculate body, inferior colliculus, hippocampus and auditory cortex (Kujawa and Liberman, 2009; Eggermont, 2017). Most previous studies believed that the auditory cortex was the most vulnerable part under noise exposure, but Cheng et al. (2016) showed that the hippocampus may be more sensitive than the auditory cortex, mainly manifested as headache, dizziness, irritability, insomnia, memory loss and even serious mental problems (Eraslan et al., 2015). Long-term noise exposure can increase the expression of corticotropin releasing-hormone (CRH) in the hippocampus and decrease the inhibition of the hypothalamic-pituitary-adrenal (HPA) axis, which may worsen depression and anxiety (Valentino et al., 2010).

In this study, we searched papers published in English and Chinese *via* PubMed, Embase, Scopus, and Web of Science database, intending to provide an overview of current knowledge relevant to the pathogenesis and susceptibility genes to NIHL.

## Pathogenesis of Noised-Induced Hearing Loss

Environmental and genetic factors can both contribute to NIHL. Environmental factors include noise intensity, noise spectrum characteristics, noise exposure time, etc. Genetic factors mainly refer to NIHL susceptibility genes. Presently, there are four main theories about the pathogenesis of NIHL, including mechanical theory, vascular theory, metabolic theory, and immunoinflammatory theory.

### Mechanical Theory

According to the mechanical theory, the internal tissue structure damage of cochlea caused by noise with over 130 dB intensity is mainly attributed to the mechanical damage (Patterson and Hamernik, 1997). High intensity noise impacts the Corti’s organ and forms a strong liquid eddy current in the cochlear duct, which can cause the rupture of the vestibular membrane and lead to the fusion of endolymph and perilymph. The cytotoxic K^+^ in endolymph could reach the tympanic scala through the orifice of the cupula cochleae and then reach the lymphatic space of the Corti’s organ, where the contact of K^+^ with hair cells leads to the destruction of cochlear sensory epithelial cells, atrophy of stria vascularis and degeneration of auditory nerve fibers. The other ways of mechanical injury were the rupture of the reticular laminae of the basilar membrane or the separation of the stereocilium of the outer hair cells from the cuticular plate, which can cause the K^+^-rich endolymph to come into contact with the hair cells. In more serious cases, noise-induced mechanical force can also cause the Corti’s organ to peel off from the basilar membrane (Spoendlin and Brun, 1973; Rajguru, 2013).

### Vascular Theory

Vascular theory believes that long-term strong noise exposure may lead to vasoconstriction around the cochlear sensory epithelium, swelling vascular endothelial cell, narrowing vascular lumen, slowed blood flow velocity, decrease in local blood perfusion, increase in blood viscosity, accumulation of platelets and red blood cells in capillaries, and obvious thickening of capillary walls. All of the aforementioned factors may ultimately lead to cochlear ischemia and hypoxia, resulting in decreased activity of otoprotective enzymes, accumulation of cellular metabolites in cells, and damage to cochlear hair cells and the Corti’s organ (Kim et al., 2018). Significant inner ear injury occurs when the perilymph oxygen partial pressure decreases by about 20% (Wu et al., 2014).

### Metabolic Theory

According to the metabolic theory, noise exposure could lead to extensive metabolic changes in the auditory system. Overexpression of free radicals including reactive oxygen species (ROS) and reactive nitrogen species (RNS) in cochlea leads to the formation of lipid peroxides and accelerates hair cells apoptosis (Zhang et al., 2017). The isoconstrictive vascular substances such as isoprostaglandin and 8-iso-prostaglandin F2α could also be released from cochlear vascular system and the Corti’s organ (Honkura et al., 2016). Strong noise exposure results in abnormal influx of K^+^ ions, leading to depolarization of membrane potential and abnormal influx of Ca^2+^, which is termed as calcium overload (Vicente-Torres and Schacht, 2006; Wang et al., 2007a). Excitotoxicity caused by large amount of glutamate release leads to edema and vacuolation of inner hair cells, neurotrophic factor deficiency, and mitochondrial dysfunction, inducing acute hair cell damage (Fridberger et al., 1998). Besides, cytokines and chemokines such as tumor necrosis factor (TNF-α), IL-6 and IL-1β are upregulated, which make contributions to the cascading amplification of exogenous and endogenous apoptotic signaling pathways, promoting the release of pro-apoptotic proteins, leading to the activation of Caspase-3, chromatin concentration, and DNA damage. Le Prell et al. (2007) believed that metabolic injury played a key role in the pathogenesis of NIHL.

### Immunoinflammatory Theory

Macrophages are the main natural immune cells in the cochlea and are important drivers of inflammation and tissue repair after noise exposure. In normal condition, cochlear macrophages inhabit spiral ligaments, spiral ganglion, basilar membrane and stria vascularis (Shi, 2010). The distribution, phenotype, number, morphology and functional state of cochlear macrophages were significantly changed after noise exposure (He W. et al., 2020). Due to the existence of tight junctions, the infiltration of macrophages and monocytes into the scala media is mainly confined to the scala tympani cavity beneath the basilar membrane, avoiding the damage and apoptosis of hair cells (Frye et al., 2018). After noise exposure, signaling pathway such as Toll-like receptor 4 (TLR-4)/nuclear factor kappa-B (NF-κB) and mitogen-activated protein kinase (MAPK)/c-Jun N-terminal kinase (JNK) were activated in cochleae, leading to upregulation of downstream inflammatory factors and chemokines including TNF-α, IL-6, IL-12, IL-1β, intercellular cell adhesion molecule-1 (ICAM-1), and monocyte chemoattractant protein-1 (MCP-1) (Wang et al., 2007b; Zhang G. et al., 2019). The release of theses cytokines and chemokines set off chain inflammatory reactions (Frye et al., 2019).

## Differences in Individual Susceptibility to Noised-Induced Hearing Loss

Noised-induced hearing loss (NIHL) is ranked as the highest incidence of industrial injury in the United States (Ralli et al., 2017), where 16% adult hearing loss are caused by exposure to industrial noise. While NIHL is a typical type of hearing loss, the causes of it are attributable to both environmental and genetic factors. Long-term exposure to noise is a prominent environmental factor for NIHL, but some studies found that not every worker who exposed to the same level of noise would develop NIHL, and the severity of NIHL also varies greatly (Irion, 1981; Hood, 1987). Taylor et al. (1965) detected the hearing threshold of workers in a textile factory and found that the workers with similar length of service had different hearing threshold which ranged from 10 to 70 dB.

In recent years, with studies of large-scale samples, it is known that even for the subjects exposed to the noise environment with similar density and duration, their hearing threshold shifts has significant individual differences (Lu et al., 2005). It reveals that there is a great difference in the susceptibility to NIHL among the population.

## Research Methods of Noised-Induced Hearing Loss Susceptible Gene

After a comprehensive analysis of some experimental studies, we summarized the methods of population research for NIHL susceptible genes as follows: sufficient number of subjects with history of noise exposure were selected as the research object, strict inclusion criteria were established, and the population whose hearing threshold locates higher than 25dB was recruited into case group, whose hearing thresholds was less than or equal to 25dB was selected into control group. The candidate genes of the two groups were detected by implementing case-control study.

There are mainly three methods for the selection of candidate NIHL genes: (1) selection of genes that have been preliminarily confirmed in animal models (2) selection of susceptibility genes that have been reported in other types of deafness; and (3) according to the pathogenic mechanism of NIHL, detect relevant genes in the corresponding pathways.

At present, the techniques for detecting susceptible genes include microarray chip, polymerase chain reaction (PCR) -restriction enzyme digestion, quantitative reverse transcription PCR, amplification refractory mutation system (ARMS)-PCR, high-throughput sequencing, and whole-exome sequencing (WES) etc. Genetic screening was carried out and compared between the two population to determine the susceptible genes which might have important influence on the pathogenesis and development of NIHL.

## Susceptibility Genes of Noise-Induced Hearing Loss

**FIGURE 1:**
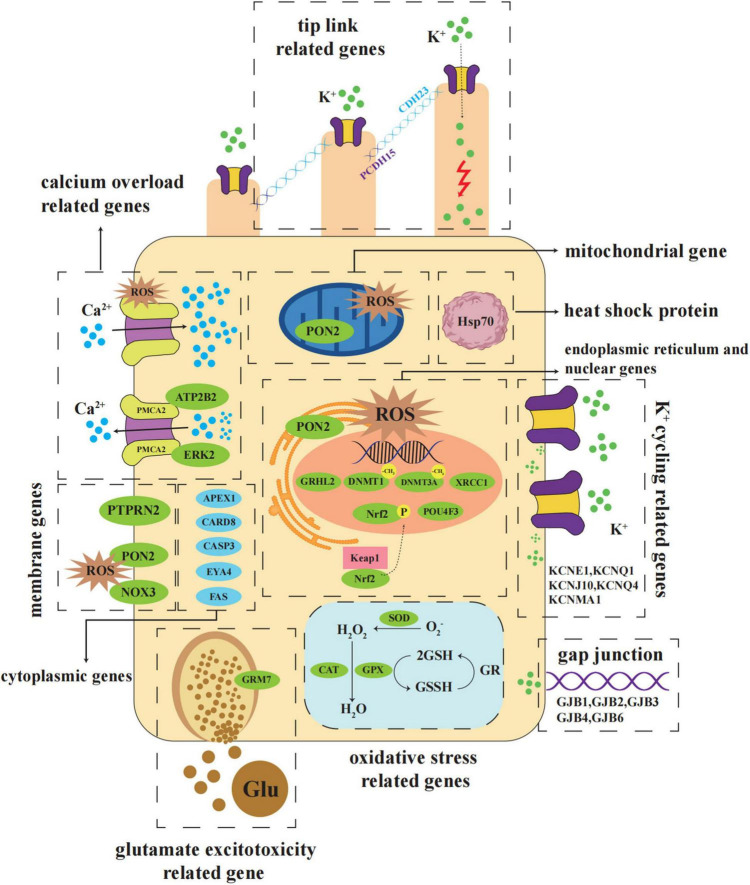
Schematic diagram of major NIHL susceptible genes distributed in the hair cells. NIHL susceptible genes are involved in the progression of oxidative stress, potassium ion cycling, calcium overload, glutamate excitotoxicity, DNA damage repair, apoptosis, and other biochemical processes. They are distributed in various locations in cells, including membrane, cytoplasm, nucleus, mitochondria, and endoplasmic reticulum. Abbreviations: Glu, Glutamate; GR, Glutathione reductase; GSH, Glutathione; GSSH, Glutathione oxidized; Keap1, Kelch-like ECH associated protein 1; PMCA2, Plasma membrane calcium-transporting ATPase 2; ROS, Reactive oxygen species.

According to the pathogenesis of NIHL, recent studies have revealed a large group of genes that are linked to NIHL involving oxidative stress, potassium ion cycling, cilia structure, heat shock protein genes 70, DNA damage repair, apoptosis, monogenic NIHL genes and others. The distribution of major susceptibility genes and the functions they are involved in is shown in Figure 1. The summary of NIHL susceptible genes and their locus is concluded in Table 1.

**TABLE 1:** Summary of NIHL susceptible genes and their locus.

Groups of genes	Gene	Full name	Genetic locus	References
Antioxidant genes	*APEX1*	Apurinic/Apyrimidinic endodeoxyribonuclease 1	rs1130409, rs1760944	Shen et al., 2016; Ding et al., 2019
	*ATP2B2* *(PMCA2)*	ATPase plasma membrane Ca^2+^ transporting 2	rs1719571, rs3209637, rs14154	Kozel et al., 2002; Li X. et al., 2016; Yan et al., 2013; Zhang S. et al., 2019
	*CAT*	Catalase	rs769217, rs208679, rs7943316, rs769214, rs475043, rs12273124, rs494024, rs564250	Konings et al., 2007; Xia et al., 2011; Yang et al., 2015; Li T. et al., 2020
	*GPX1*	Glutathione peroxidase 1	rs1987628	Wen et al., 2014; Li J. Y. et al., 2020
	*GST*	Glutathione S-transferase	rs1695, rs1049055, rs10712361	Lin et al., 2009; Shen et al., 2012; Loukzadeh et al., 2019; Zong et al., 2019; Li Y. H. et al., 2020
	*NFE2L2 (NRF2)*	Nuclear factor erythroid 2-related factor 2	rs77684420, rs6726395, rs1962142, rs6721961	Honkura et al., 2016; Wang et al., 2019
	*NOX3*	NADPH Oxidase 3	rs12195525, rs33652818	Lavinsky et al., 2015; Xin et al., 2021
	*PON2*	Paraoxonase 2	rs12026, rs7785846, rs12704796, rs987539, rs7493, rs7786401	Cao et al., 2013; Li X. et al., 2016; Bhatt et al., 2020; Wu et al., 2020; Zhou H. et al., 2020
	*SOD1*	Superoxide dismutase 1	rs2070424, rs10432782	Liu et al., 2010b
	*SOD2*	Superoxide dismutase 2	rs4880, rs2855116	Ohlemiller et al., 1999; Fortunato et al., 2004; Liu et al., 2010a; Wang et al., 2014; Wang J. et al., 2017
Potassium ion cycling related genes	*KCNQ1*	Potassium voltage-gated channel subfamily Q member 1	rs800336, rs2056892, rs2011750, rs2283158, rs2283179, rs2283205, rs231899, rs760419, rs163171, rs8234, rs7945327, rs11022922, rs718579, rs463924	Van Laer et al., 2006; Pawelczyk et al., 2009; Ding et al., 2020
	*KCNQ4*	Potassium voltage-gated channel subfamily Q member 4	rs34287852, rs2769256, rs727146, rs4660468, rs12143503, rs4660470	Van Laer et al., 2006; Pawelczyk et al., 2009; Guo et al., 2018b; Zhou W. H. et al., 2020
	*KCNE1*	Potassium voltage-gated channel subfamily E regulatory subunit 1	rs915539, rs2070358, rs1805127, rs1805128	Van Laer et al., 2006; Ding et al., 2020
	*KCNJ10*	Potassium voltage-gated channel subfamily J member 10	rs1130183, rs1186675	Van Laer et al., 2006; Pawelczyk et al., 2009; Bhatt et al., 2020
	*KCNMA1*	Potassium calcium-activated channel subfamily M alpha 1	rs696211, rs1436089	Konings et al., 2009b; Zhang X. et al., 2019
	*GJB1 (Cx32)*	Gap Junction Protein Beta 1	rs747181, rs1997625	Van Laer et al., 2006; Pawelczyk et al., 2009
	*GJB2 (Cx26)*	Gap Junction Protein Beta 2	rs3751385, rs5030700, rs137852540	Van Laer et al., 2006; Pawelczyk et al., 2009; Zhou et al., 2016
	*GJB3 (Cx31)*	Gap Junction Protein Beta 3	rs476220	Van Laer et al., 2006
	*GJB4 (Cx30.3)*	Gap Junction Protein Beta 4	rs1998177, rs755931	Van Laer et al., 2006; Pawelczyk et al., 2009
	*GJB6 (Cx30)*	Gap Junction Protein Beta 6	rs945370, rs2065796, rs2065797	Van Laer et al., 2006
	*SLC12A2*	Solute carrier family 12 member 2	rs1962291, rs1560637, rs790153, rs790156, rs10089	Van Laer et al., 2006
Cilia structure related genes	*CDH23*	Cadherin related 23	rs1227049, rs1227051, rs3802711, rs3752752, rs41281334	Yang et al., 2006; Kowalski et al., 2014; Yu et al., 2016; Bhatt et al., 2020; Jiao et al., 2020; Jiang et al., 2021
	*PCDH15*	Protocadherin related 15	rs11004085, rs7095441, rs1100085, rs10825122, rs1930146, rs2384437, rs4540756, rs2384375	Konings et al., 2009b; Xu et al., 2017a,b
	*MYH14*	Myosin heavy chain 14	rs667907, rs588035	Konings et al., 2009b; Fu et al., 2016
Heat shock protein genes 70	*HSPA1A*	Heat shock protein family A member 1A	rs1043618, rs1061581	Li et al., 2017
	*HSPA1B*	Heat shock protein family A member 1B	rs2763979	Konings et al., 2009a; Chang et al., 2011
	*HSPA1L*	Heat shock protein family A member 1L	rs2075800, rs2227956	Chang et al., 2011; Li Y. H. et al., 2016; Li et al., 2017
DNA damage repair related genes	*DNMT1*	DNA methyltransferase 1	rs2228611	Guo et al., 2018a
	*DNMT3A*	DNA methyltransferase 3 alpha	rs749131, rs1550117	Guo et al., 2018a
	*EYA4*	EYA transcriptional coactivator and phosphatase 4	rs3777781, rs212769, rs3813346, rs9321402, rs9493627	Zhang et al., 2015; Yang Q. et al., 2016; Yang et al., 2017
	*OGG1*	8-Oxoguanine DNA glycosylase	rs1052133	Shen et al., 2014
Apoptosis related genes	*CASP3*	Caspase 3	rs1049216, rs6948	Wu et al., 2017
	*ERK2* *(MAPK1)*	Extracellular signal-regulated kinase 2	Null (animal experiment)	Kurioka et al., 2015
	*JNK1* *(MAPK8)*	C-Jun N-terminal kinases 1	rs11598320, rs8424	Sun et al., 2021
Other NIHL susceptible genes	*AUTS2*	Activator of transcription and developmental regulator	rs35075890	Niu et al., 2021
	*CARD8*	Caspase recruitment domain family member 8	rs2043211	Miao et al., 2021
	*DFNA5* *(GSDME)*	Gasdermin E	rs2521758	Zhang et al., 2015
	*FAS*	Fas cell surface death receptor	rs1468063, rs2862833	Xu et al., 2021
	*FOXO3*	Forkhead box O3	rs2802292, rs10457180, rs12206094	Guo et al., 2017, Guo et al., 2018c; Jiao et al., 2017
	*GAPDH*	Glyceraldehyde-3-phosphate dehydrogenase	rs6489721	Wan et al., 2020
	*GRHL2*	Grainyhead like transcription factor 2	rs3735715, rs1981361, rs666026, rs611419	Li X. et al., 2013; Zhang et al., 2015; Xu et al., 2016; Yang Q. Y. et al., 2016; Li X. et al., 2020
	*GRM7*	Glutamate metabotropic receptor 7	rs1485175, rs1920109, rs9826579	Yu et al., 2018a,b
	*HDAC2*	Histone deacetylase 2	rs10499080, rs6568819	Wang et al., 2021
	*HOTAIR*	HOX transcript antisense RNA	rs4759314	Wang B. et al., 2017
	*IL-6*	Interleukin 6	rs1800795	Braga et al., 2014
	*ITGA8*	Integrin subunit alpha 8	rs10508489	Xia et al., 2011
	*NCL*	Nucleolin	rs7598759	Grondin et al., 2015
	*NOTCH1*	Notch receptor 1	rs3124594, rs3124603	Ding et al., 2018
	*NRN1*	Neuritin 1	rs3805789	Liu et al., 2021
	*PER1*	Period circadian regulator 1	rs2585405	Chen et al., 2021
	*POU4F3*	POU class 4 homeobox 3	rs1368402, rs891969	Xu et al., 2016
	*PTPRN2*	Protein tyrosine phosphatase receptor type N2	rs10081191	Niu et al., 2021
	*SIK3*	Salt-inducible kinase 3	rs493134, rs6589574, rs7121898	Yin et al., 2020
	*STAT3*	Signal transducer and activator of transcription 3	rs1053005	Gao et al., 2021
	*TSP*	Thrombospondin	Null (animal experiment)	Smeriglio et al., 2019
	*UBAC2*	UBA domain containing 2	rs3825427	Wan et al., 2022
	*WHRN*	Whirlin	rs12339210	Jiang et al., 2021
	*XPO5*	Exportin 5	rs11077	Wang et al., 2020
	*XRCC1*	X-Ray repair cross complementing 1	rs1799782	Ding et al., 2019

### Antioxidant Genes

According to the metabolic theory, oxidative stress plays a major role in the pathomechanisms of NIHL (Spoendlin and Brun, 1973; Rajguru, 2013; Chen et al., 2020). Mutations of oxidative stress related genes would disturb the balance of the oxidative and antioxidative system in the cochlea, thus fail to eliminate the oxidative damage of ROS, leading to the structural and functional disorders of the cochlea which ultimately result in hearing loss.

#### ATPase Plasma Membrane Ca^2+^ Transporting 2 (ATP2B2, PMCA2)

*ATP2B2*, encoding plasma membrane calcium-transporting ATPase isoform2 (PMCA2), is located on human chromosome region 3p25, and played an important role on intracellular calcium homeostasis (Yang et al., 2013). In an animal experiment, Kozel et al. (2002) hypothesized that *Atp2b2*^+/–^ mice may be more susceptible to NIHL. Recently, Li X. et al. (2016) designed a study to investigate whether genetic variability in *ATP2B2* was associated with high susceptibility to NIHL in Chinese Han nationality population. However, no significant main effect was observed for *ATP2B2* gene single-nucleotide polymorphisms (SNPs) (rs1719571, rs3209637 and rs4327369) in their study because of the small sample size. In another case-control study of 760 Chinese textile workers, the results indicated that the rs3209637 C genotype of *ATP2B2* may lead to a greatly increased incidence of NIHL. Meanwhile, the analysis also demonstrates that *ATP2B2* SNPs (rs1719571, rs14154, and rs3209637) have a great effect on NIHL (Zhang S. et al., 2019).

#### Catalase

Catalase (CAT) is a ubiquitous enzyme in all organisms, functioning as a key antioxidant enzyme in the defense against oxidative stress. Catalase encoded by *CAT* gene can decompose hydrogen peroxide (H_2_O_2_), maintain the balance of redox in the body, and reduce the oxidative damage of cochlea caused by oxidative stress. Yang et al. (2015) screened 719 unrelated Chinese Han adults, including 225 healthy volunteers and 494 noise-exposed workers, and found that rs208679 and rs769217 SNPs were significantly associated with the susceptibility to NIHL. For rs208679 recessive effect, GG genotype showed significantly augmented risk when exposing to noise less than 85dB, while for rs769217 dominant effect, TT/TC combined genotypes significantly increased the risk of NIHL when noise intensity was between 85dB-92dB.

#### Glutathione Peroxidase 1

The GPX protein belongs to the glutathione peroxidase (GPx) family, which reduces H_2_O_2_ and organic hydroperoxides originated from Fenton and Haber Weiss reactions coupling with other glutathione (GSH) and GSH reductase redox systems (Evans and Halliwell, 1999). GPx oxidizes GSH into glutathione oxidized (GSSH), while glutathione reductase (GR) reduces GSSH into GSH. Moreover, H_2_O_2_ is catalyzed and broke down into H_2_O by GPx and CAT to achieve antioxidant effects (Figure 1). Ohlemiller et al. (2000) performed research to investigate the association between cellular *Gpx1* gene and the susceptibility to NIHL in mice. The significant results revealed that *Gpx*-deficient mice showed increased susceptibility to NIHL. Wen et al. (2014) scrutinized the relationship between SNPs of GPX1 gene rs3448, rs1050450, rs1800668, and rs1987628, and the risk of developing NIHL among Chinese Han population. They clarified that GPX1 SNP rs1987628 may be a risk factor of NIHL. Another study of a limited sample set using genotyping kit to analyze the SNPs discovered that the individuals carrying rs1987628 GA genotype of GPX1 had a higher NIHL risk than those carrying the GG genotype (Li J. Y. et al., 2020).

#### Glutathione S-Transferase

Glutathione S-transferase (GST) can catalyze the binding of a variety of endogenous or exogenous compounds to reduced glutathione, which serves as an important protective antioxidant factor in the cochlea. Shen et al. (2012) analyzed the polymorphism of *GST* gene in 444 workers with NIHL and 445 workers with normal hearing to find out the relationship between the polymorphism and the susceptibility to NIHL. The results showed that null genotype of *GSTM1* rs10712361 had a higher risk of NIHL comparing with wild-type genotype. Lin et al. (2009) found that individuals carrying all genotypes with *GSTM1* null, *GSTT1* null, and *GSTP1 lle* (Guo et al., 2017)/*lle* (Guo et al., 2017) were more susceptible to NIHL.

#### Nuclear Factor Erythroid 2-Related Factor 2

*NRF2*, existing widely in tissues, is a key transcription factor in the regulation of oxidative stress. When affected by oxidative stress, NRF2 dissociates from Kelch like epichlorohydrin associated protein 1 (Keap1), a negative regulator of NRF2, and is transferred to the nucleus to recognize and bind antioxidant response elements (ARE) (Sivandzade et al., 2019). Thus, the transcription of downstream antioxidant enzyme genes is initiated, including heme oxygenase 1 (*HO-1*), superoxide dismutase (*SOD*), triphosphopyridine nucleotide (*NADPH*), *GST, GR* and *GPx* (He F. et al., 2020). Honkura et al. (2016) explored the contribution of *Nrf2* to cochlear protection *via Nrf2^–/–^* mice models. They found that *Nrf2* deficiency could exacerbate NIHL as auditory brainstem response (ABR) threshold shifts of the *Nrf2^–/–^* mice was significantly larger than the wild-type mice at 7 days post-exposure. Although noise exposure does not obviously change the expression of *Nrf2* target genes, the potent NRF2-activating drug, CDDO-Im used before the noise exposure could preserve the integrity of hair cells and improve post-exposure hearing level. Wang et al. (2019) found that persons with a G allele (*NRF2* tagSNP rs6726395) in addition to rs77684420 and the rs6726395, rs1962142, rs6721961, and rs77684420 haplotype had associations that may be more susceptible to NIHL.

#### Triphosphopyridine Nucleotide Oxidase-3

The NOX family of ROS-generating NADPH oxidases consists of 7 members: NOX1 to NOX5, DUOX1 and DUOX2. In particular, NOX3 is almost exclusively expressed in the inner ear, and it has been demonstrated to generate superoxide constitutively which is converted to H_2_O_2_ by SOD, which can in turn participate in cell signaling events (Krause, 2004; Forman et al., 2010). In a previous study, a significant reduction in the intensity of *NOX3* immunolabeling was observed in the inner sulcus region of the cochlea after noise exposure, and down-regulation of *NOX3* may represent an endogenous protective mechanism to reduce oxidative stress in the noise-exposed cochlea (Vlajkovic et al., 2013). Xin et al. (2021) conducted a case-control study in five factories in China, and illustrated the association between rs12195525 and NIHL susceptibility. For further exploration, Lavinsky et al. (2015) verified that *Nox3* is involved in NIHL susceptibility in *Nox3*^het^*/Nox3*^het^** and *Nox3*^het^*/* + mutant mice, which was frequency specific at 8 kHz. Besides, the significant and highly potential association of rs33652818 with ABR at 8 and 4 kHz was observed.

#### Paraoxonase-2

*PON2* gene, localized in endoplasmic reticulum (ER), mitochondria and nuclear envelope, is involved in the process of defending ROS, ER stress, mitochondrial superoxide formation, and apoptosis (Altenhofer et al., 2010; Witte et al., 2011). Li X. et al. (2016) studied the polymorphisms of rs12026, rs7785846, and rs12704796 in *PON2* in 221 patients with NIHL and 233 subjects with normal hearing by logistic regression analysis. It was found that rs12026 CG and CG + GG genotypes and rs7785846 CT and CT + TT genotypes were highly susceptible to NIHL. Wu et al. (2020) confirmed these results that *PON2* gene affects the NIHL susceptibility of cochlea.

#### Superoxide Dismutase 1 and 2

Superoxide Dismutase (SOD) is an important antioxidant enzyme in organisms and the primary substance for scavenging ROS in the body, which is involved in the reaction of superoxide anion (O_2_^–^) and H^+^ to produce H_2_O_2_. It plays an important role on blocking cell damages caused by ROS and repairing the damaged cells in time. Liu et al. (2010a,b) analyzed the audiometric data of 2400 Chinese Han people exposed to occupational noise, and selected the 10% most susceptible and the 10% most resistant individuals as subjects to collect DNA samples. It has been found that the *SOD1* AA genotype at the rs2070424 was protective against NIHL, while the *SOD1* GG genotype of rs10432782 and the CT genotype of rs4880 (*SOD2* V16A SNP) was associated with higher occurrence of NIHL. However, the above results were not in agreement with a former research based on a Swedish population, which suggests that *SOD* genetic polymorphism may confer a race-specific contribution (Carlsson et al., 2005).

### Potassium Ion (K^+^) Cycling Related Genes

**FIGURE 2:**
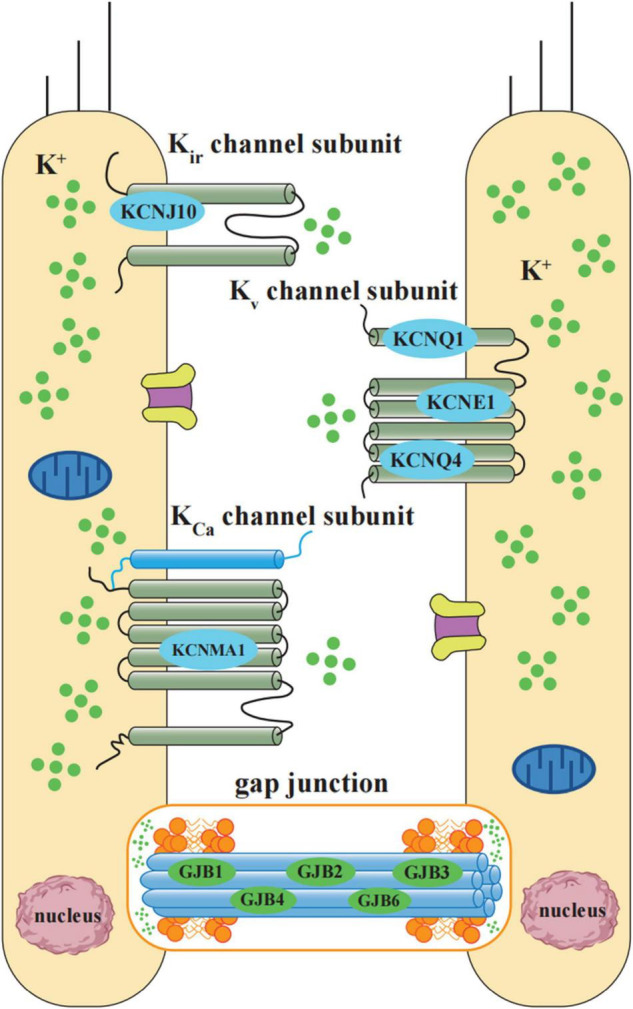
Schematic diagram of potassium ion cycling related NIHL susceptibility genes. K^+^ cycling related NIHL susceptibility genes include K^+^ channel proteins and gap junction proteins. According to the operational mechanism and structures, NIHL susceptibility related K^+^ channels can be classified into 3 groups: inward rectifier (K_ir_, including *KCNJ10*), voltage-gated (K_v_, including *KCNQ1*, *KCNE1*, and *KCNQ4*), and Ca^2+^activated (K_Ca_, including *KCNMA1*). Gap junctions between hair cells and non-sensory cells are primarily formed by a family of connexin proteins, which is encoded by gene *GJB1*, *GJB2*, *GJB3*, *GJB4*, and *GJB6.* Gap junction-mediated intercellular communication plays an essential role in K^+^ exchange.

As an important charge carrier in the process of sound sensory conduction, K^+^ can be secreted to the endolymph, and then utilized by the sensory hair cells of the inner ear through the mechanically sensitive K^+^ channel, and this ion circulation ensures the generation of hearing. The related genes which has been proved susceptible to NIHL are illustrated in Figure 2.

#### Potassium Voltage-Gated Channel Subfamily E Regulatory Subunit 1 and Potassium Voltage-Gated Channel Subfamily Q Member 1

*KCNE1* encodes a regulatory subunit of the KCNQ1 potassium channel-complex. Both KCNE1 and KCNQ1 are necessary for normal hearing. Pawelczyk et al. (2009) performed a study to clarify the hypothesis that genetic variability in genes of the potassium recycling pathway may be a risk factor for the development of NIHL. The significant results revealed that the AA genotype in rs2070358 appeared more frequently in resistant individuals than in susceptible ones, while genotype GG was more often among susceptible subjects. Recently, another study (Ding et al., 2020) was designed to investigate the association between genetic mutations in the *KCNE1* gene and susceptibility to NIHL in the Chinese population. Their results showed that the rs3453 C allele and the rs1805127 G allele were associated with increased susceptibility to NIHL.

#### Potassium Voltage-Gated Channel Subfamily Q Member 4

Potassium Voltage-Gated Channel Subfamily Q Member 4 (KCNQ4) is a voltage-gated potassium channel that plays essential roles on maintaining ion homeostasis and regulating hair cell membrane potential. Guo et al. (2018b) conducted a genetic association study to scrutinize the association between *KCNQ4* polymorphism and susceptibility to NIHL. They detected that rs4660468 CT/TT genotype and T allele may increase the susceptibility. In another study among Chinese population, the SNPs of rs4660468, rs4660470, rs34287852 in *KCNQ4* were genotyped by Zhou W. H. et al. (2020). They identified that the risk of developing NIHL in subjects carrying TA genotype of rs4660470 was 2.197 times than the one carrying TT genotypes, suggesting that the mutant allele A of rs4660470 in *KCNQ4* may be a risk factor for developing NIHL.

#### Potassium Inwardly Rectifying Channel Subfamily J Member 10

*KCNJ10* encodes the inward-rectifying potassium channel that is expressed in the brain, the inner ear, and kidney. Pawelczyk et al. (2009) conducted a study to explore the putative hypothesis that genetic variations in ten genes associated with the potassium recycling pathway in the inner ear may influence susceptibility to the development of NIHL. Their results discovered that the polymorphism of rs1130183 in *KCNJ10* may be a risk factor for the development of NIHL. In addition, Bhatt et al. (2020) performed research to investigate the relationship between candidate genetic variants and NIHL in young musicians, they also identified that *KCNJ10* rs1130183 showed significant association with the distortion product otoacoustic emission (DPOAE) signal-to-noise ratio (SNR) in the right ear.

#### Gap Junction Protein Beta 2 (Connexin 26, Cx26)

*GJB2*, encoding a gap junction protein expressed in the inner ear, has been considered to be involved in the potassium recycling pathway in the cochlea. Van Eyken et al. (2007) performed a study to investigate the association between the *GJB2* 35delG mutation and the development of NIHL. Frustratingly, the results suggested that 35delG carriers had no increased susceptibility to the development of NIHL. However, in an animal study, Zhou et al. (2016) established a *Connexin26* knockdown mouse model to investigate the relationship between *Connexin26* gene and NIHL. Their results indicated that decreased *Connexin26* expression may contribute to the increased susceptibility to NIHL and promote the cell degeneration in the Corti’s organ.

### Cilia Structure Related Genes

Tip links of the hair cells play a crucial role in the process of mechano-electrical transduction (MET), transforming the mechanical sound stimuli into electrical signals (Sakaguchi et al., 2009). The main constituent of tip links are cadherin related 23 (CDH23) and procadherin related 15 (PCDH15), atypical members of the cadherin superfamily. Cadherin is a calcium-dependent cellular adhesion glycoprotein, which plays an important role in cell recognition, migration, tissue differentiation, the composition of adult tissues and embryonic development. The polymorphism of those genes is closely related to the susceptibility to NIHL. Besides, the damage of MYH14, located at the tip links between hair cells and hair cells, hair cells and supporting cells, also leads to susceptibility to NIHL.

#### Cadherin Related 23

Cadherin Related 23 (CDH23) is an important protein which is mainly expressed in the cilia of inner hair cells and vestibular membrane (Wilson et al., 2001). Anchored to ciliated microfilaments by actin, it forms a protein network with myosin VIIA for functional activity (Boeda et al., 2002). Its primary function is to maintain the structure and function of hair cell cilia and the ion composition of endolymph, which ensure the mechanical-electrical conversion of sound waves can be carried out normally during the transduction of sound waves in the inner ear (Siemens et al., 2004). It was evidenced in adult mice that *Cdh23* mutant mice were susceptible to NIHL. The results showed that the threshold of compound action potential (CAP) was increased by about 50dB at 12 kHz and 30 kHz frequency, which was more than twice that of wild type mice (Holme and Steel, 2004). Kowalski et al. (2014) selected 314 workers with the worst hearing as the experimental group and 313 workers with the best hearing as the control group from 3860 workers database exposed to the same noise environment. Statistical analysis showed that the genotype of the SNP rs3752752 located in exon 21 was closely related to NIHL susceptibility, in which CC genotype was more common in susceptible population, while CT genotype appeared more frequently in the group with better noise tolerance. Another study (Yang et al., 2006) revealed that individuals with rs3802721TT genotype, rs1227049CC genotype and GG genotype at the end of exon 7 were more susceptible to NIHL.

#### Procadherin Related 15

*PCDH15* encodes a membrane protein that mediates calcium-dependent cell adhesion. It is considered that tip-link is composed of proteins encoded by *PCDH15* and *CDH23* genes (Rowlands et al., 2000). The protein encoded by the *PCDH15* forms the lower part of the tip-link, and the CDH23 forms the upper part. *In vitro*, the extracellular components of PCDH15 and CDH23 form parallel homodimers, and the homodimers are arranged in a Ca^2+^ dependent antiparallel manner (Ahmed et al., 2006). In recent years, it has been found that there is a correlation between *PCDH15* gene polymorphism and NIHL susceptibility. Zhang et al. (2014) selected 476 workers with NIHL and 475 workers with normal hearing from a factory in China for a case-control study. There is no difference in sex ratio, noise exposure years and exposure intensity between the two groups. It was found that the allele frequency and genotypes of rs1104085 were significantly correlated with NIHL susceptibility, that is, the susceptibility of variant allele CT or CC genotype was significantly lower than that of wild type TT homozygotes. Besides, SNPs of rs1100085, rs10825122, rs1930146, rs2384437, rs4540756, and rs2384375 were also found to have correlations with NIHL.

#### Myosin Heavy Chain 14

The *MYH14* is located on chromosome 19 and encodes myosin-binding protein C. It is an ATP-dependent molecular motor involved in cytoskeletal rearrangement and ion gate control. MYH14 was first identified as the causative gene for neurogenic deafness in 2004 (Donaudy et al., 2004). Konings et al. (2009a) conducted an association study of NIHL based on a candidate gene approach. They found two SNPs in *MYH14* (rs667907 and rs588035) that resulted in significant associations in the Polish sample set and significant interactions with noise exposure level in the Swedish sample set. Fu et al. (2016) established *Myh14* knockout mice using CRISPR/Cas9 technology and clarified the role of *MYH14* in the cochlea and NIHL. They found that *Myh14*^–/–^ mice were more susceptible to high-intensity noise compared to control mice. After acoustic trauma, more pronounced loss of outer hair cells was observed in *Myh14*^–/–^ mice than in wild-type controls, suggesting that *Myh14* may play a beneficial role in protecting the cochlea after acoustic overstimulation in CBA/CaJ mice.

### Heat Shock Protein Genes 70

Heat shock protein genes (HSPs) can be overexpressed in the inner ear by stimulation such as physiological stress, ototoxic drugs, high temperature and noise. Among them, HSP70 is a dominant type of heat stress protein which has great protective effect. Gratton et al. (2011) observed the difference of cochlear membrane labyrinth gene expression between noise-susceptible experimental group and noise-tolerant control group. It was found that the protein contents of HSP70 and HSP40 in the control group were significantly higher than those in the experimental group, indicating that the expression of *HSP70* gene may play an important role on protecting animals from NIHL. Lei et al. (2017) used Meta analysis to comprehensively analyze the relationship between *HSP70* polymorphism and NIHL susceptibility, and concluded that the polymorphism of rs1061581 and rs2227956 may be closely related to the susceptibility to NIHL. Li et al. (2017) screened 286 NIHL patients by measuring the hearing threshold of iron and steel workers, and selected another 286 normal hearing workers in the same noise environment as the control group. It was found that the proportion of TT genotype of rs2763979 in Chinese Han population was higher than that of CC/TC genotype in the NIHL group.

### DNA Damage Repair Related Genes

#### Eyes Absent Homolog 4

Eyes Absent Homolog 4 (EYA4) is a member of the eye absent family of proteins that encode transcriptional activator-related proteins and plays an important role on regulating tissue-specific differentiation during embryonic development (Borsani et al., 1999). It also participates in a variety of biological activities including maintaining the development and maturation of the Corti’s organ (Wayne et al., 2001). Zhang et al. (2015) investigated the relationship between the polymorphisms of *EYA4* and the risk of developing NIHL. The results of this study showed that rs3777781 and rs212769 in the *EYA4* gene were significantly associated with the risk of NIHL. In rs3777781, carriers of the AT and AA genotypes had a reduced risk of NIHL compared to subjects carrying the TT genotype. In rs212769, carriers of the AG and AA genotypes had an increased risk of NIHL compared to subjects with the GG genotype. In another case-control study (Yang et al., 2017), subjects carrying the rs3813346 TT genotype had a higher risk of NIHL than subjects carrying the GG genotype in the noise intensity > 85 dB group. In contrast, in the cumulative noise exposure (CNE) > 98 dB-year group, haplotype CGT showed a protective role in the development of NIHL compared to haplotype TGC, suggesting that genetic polymorphisms in the *EYA4* gene may be a genetic susceptibility factor for NIHL.

#### 8-Oxoguanine DNA Glycosylase

Human 8-hydroxyguanine glycosylase (hOGG1) is a DNA repair enzyme in the base excision repair pathway, whose main function is to recognize and excise 8-oxo G in the DNA double strand and repair damaged DNA. Shen et al. (2014) designed research to investigate the relationship between the gene polymorphism (*hOGG1* Ser326Cys) of rs1052133 and susceptibility to high frequency hearing loss. The *hOGG1* Cys/Cys genotype was found to be a possible risk factor for high-frequency hearing loss, and stratified analysis revealed it was also associated with risk factors such as years of work in noisy jobs, noise exposure level and smoking. Thus, they concluded that the *hOGG1* Cys/Cys genotype may be a risk factor for high frequency hearing loss in the Chinese Han population.

### Apoptosis Related Genes

#### Extracellular Signal-Regulated Kinase 2

Extracellular signal-regulated kinase (ERK) is a member of the MAPK cascades which is a key signaling pathway that control a multitude of cellular processes such as cell survival, protein synthesis, cell proliferation, growth, migration, and apoptosis (Cargnello and Roux, 2011). Recently, accumulative evidences indicate that ERK is involved in response to cellular stress such as noise exposure. When activated by stimulation, ERK2 transfers from the cytoplasm to the nucleus, result in the activation of downstream transcription factors who would further execute kinds of cellular functions (Seger et al., 1991). Kurioka et al. (2015) revealed that conditional *Erk2* knockout mice were more susceptible to noise damage and had slower recovery from NIHL compared to control mice. Furthermore, they detected a significant lower survival rate of inner hair cells in *Erk2* knockout mice. Their results suggest that *Erk2* is essential to the survival of hair cells in NIHL. However, to the best of our knowledge, the research concerning *ERK2* polymorphisms in NIHL population is nearly a piece of blank.

#### C-Jun N-Terminal Kinases 1

C-Jun N-terminal kinase (JNK), also known as stress-activated protein kinase (SAPK), is a member of the MAPK family (Hollville et al., 2019). The JNK stress pathways are involved in many different intracellular signaling pathways that control diverse cellular processes such as cell growth, differentiation, transformation, and most importantly, apoptosis (Zeke et al., 2016). Sun et al. (2021) conducted a study to explore the effect of *JNK1* polymorphisms on the sensitivity of NIHL, and the results indicated that the rs11598320 TT genotype and the rs8428 TT genotype may be associated with a higher risk of NIHL. Interestingly, a previous study has also reported that prednisone, a well-known steroid clinically used in the treatment of hearing loss, could inhibit the IL-1β-induced activation of *JNK1* (Hong and Jang, 2014).

### Other Noised-Induced Hearing Loss Susceptible Genes

#### Caspase Recruitment Domain Family Member 8

Inflammation is a complex process that is thought to contribute to the development of NIHL. CARD8 is an important component of the inflammasome and has been implicated in inflammation. Miao et al. (2021) conducted a study to investigate the relationship between *CARD8* gene polymorphisms and NIHL risk and to infer the underlying mechanisms. They verified three SNPs (rs2043211, rs1062808 and rs12459322) in a Chinese population including 610 NIHL cases and 612 normal hearing controls. The haplotype AGG (rs2043211-rs1062808-rs12459322), the AA genotype and A allele of rs2043211 were found associated with a reduced risk of NIHL.

#### Fas Cell Surface Death Receptor

Reactive oxygen species (ROS) production in the cochlea and blood caused by noise exposure leads to the processes of oxidative stress, lipid peroxidation, and DNA damage, during which *FAS* is activated. Xu et al. (2021) conducted case-control research to investigate the relationship between genetic polymorphisms in the *FAS* gene and NIHL risk. 692 NIHL workers and 650 controls were genotyped for four SNPs, among which two polymorphisms, rs1468063 and rs2862833, were associated with NIHL. Individuals harboring rs1468063-TT or rs2862833-AA genotypes had a decreased risk of NIHL.

#### Forkhead Box O3

*FOXO3* is a gene with a variety of biological functions and is closely related to mammalian longevity. It regulates specific activation of transcription factors to exert effects on cell differentiation, apoptosis, cell cycle, DNA damage repair and oxidative stress (Stefanetti et al., 2018). Through the study of the animal model of NIHL, Gilels et al. (2017) found that the outer hair cells of *Foxo3* knockout mice were more seriously damaged than those of normal mice after the same intensity of noise exposure, and the severity of hearing loss increased significantly, indicating that *Foxo3* is an important protective gene for mice to maintain hearing after noise exposure. Guo et al. (2017) conducted research to explore the effects of *FOXO3* polymorphisms on individual NIHL susceptibility. The results proved that individuals with the G allele of rs2802292, G allele of rs10457180, T allele of rs12206094 and the haplotype GAC and others (TGT/GGT/GGC/GAT) (rs2802292-rs10457180-rs12206094) are associated with an increased risk of NIHL in a Chinese population. In addition, they revealed that GT-GG genotype in *FOXO3* may be a risk factor for occupational NIHL (Guo et al., 2018c). They concluded that the genetic polymorphisms rs2802292, rs10457180, rs12206094 and rs12212067 within *FOXO3* have the potential to be biomarkers for noise exposed impairment for workers.

#### Grainyhead-Like 2

Grainyhead-Like 2 (GRHL2) is a transcription factor that expressed in epithelial tissues, it not only plays a central role in embryonic development, but also contributes to epithelial cell maintenance (Peters et al., 2002). Li X. et al. (2013) conducted a study to evaluate the contribution of the *GRHL2* polymorphisms to NIHL susceptibility in a Chinese population and found that the subjects carrying rs611419 AT/TT were more resistant to NIHL compared with those carrying the AA genotype. In addition, another study revealed that the CC genotype of rs1981361 in *GRHL2* gene was contributed to a higher risk of NIHL (Xu et al., 2016). Additionally, the fact that the rs3735715 GG genotype had a higher NIHL risk compared with the GA genotype was also verified in another study among Chinese population (Yang Q. Y. et al., 2016).

#### Metabolic Glutamate Receptor 7 Gene

Metabolic Glutamate Receptor 7 Gene (GRM7) is mainly responsible for glutamate-mediated postsynaptic excitation of neurons. In order to study the effect of *GRM7* polymorphism on NIHL susceptibility, Yu et al. (2018a) selected 292 NIHL patients and 584 workers with normal hearing in a steel factory as subjects. It is found that the C allele genotype of the rs1485175 mutant of *GRM7* gene plays an important role in reducing the incidence of NIHL. Permutation test of generalized multiple dimensionality reduction (GMDR) suggested that rs1920109, rs1485175 and rs9826579 might interact with each other in the pathogenesis of NIHL.

#### HOX Transcript Antisense RNA

LncRNA HOTAIR is a non-coding RNA that plays a crucial role in RNA processing, gene regulation, chromatin modification, gene transcription, post-transcriptional regulation (Kalwa et al., 2016). It is involved in the alterations of oxidative stress levels, cell proliferation, cell cycle progression and apoptosis. As its expression level is always dysregulated in variety of cancers, it is considered to be used as a potential biomarker (Yang et al., 2019). In order to explore the effect of *HOTAIR* polymorphisms on the NIHL susceptibility, three tag SNPs of the *HOTAIR* (rs874945, rs4759314 and rs7958904) were genotyped in a Chinese population including 570 NIHL cases and 570 controls (Wang B. et al., 2017). The results showed that individuals with the G allele of *HOTAIR* tagSNP rs4759314 and the haplotype (rs874945, rs4759314 and rs7958904) were associated with an increased risk of NIHL.

#### POU Class 4 Homeobox 3

POU Class 4 Homeobox 3 (POU4F3), also known as Bm3.1 or Bm3c, is a transcription factor which is important for the maturation, differentiation and survival of inner ear hair cells. Xu et al. (2016) performed a matched case-control study to explore the relationship between SNPs in the *POU4F3* gene and susceptibility to high frequency hearing loss in a Chinese population. They revealed that when CNE > 95 dB, individuals carrying the AA genotype had an increased risk of hearing loss compared to the CC/CA genotype at SNP rs1368402. Compared to the AA/GA genotype at rs891969, the GG genotype revealed to be a risk genotype.

## Concluding Remarks

As the death of hair cells in the cochlear is irreversible, and NIHL is a completely preventable disease, it is particularly important to prevent the potential hearing impairment in advance through possible screening and evaluation. The explore of NIHL susceptible genes offers an opportunity to decrease the incidence of hearing loss by risk assessment as early as infant. The incidence of NIHL would be significantly reduced by distributing the susceptible individuals away from intense noise exposure. For example, factories could assign different employees according to their genotype of NIHL susceptible genes to avoid the occupational impairment; NIHL susceptibility screening could also be applied during conscription.

Although dozens of possible susceptibility genes related to NIHL have been screened, there is still a big gap between practical application and researches. Taylor et al. (1965) first established a linear regression model between noise exposure and high frequency hearing threshold in 1965. It was found that the distribution of NIHL susceptibility in the population showed a unimodal left bias, and there was no single peak on the right side of the main peak (susceptible area), suggesting that the susceptibility is related to many factors and is likely to be affected by multiple minor genes, which increases the difficulty of the study on susceptibility genes.

In relation to the screening of NIHL susceptibility genes, there are some limitations whether using animal research or population study. For animal research, although it has the advantages of short test cycle and easy to obtain materials, the results must be verified in the population. For population study, family analysis is the most effective method to study susceptibility genes, but medical ethics cannot expose all subjects to noise environment, so pedigree analysis cannot be used in the study, only NIHL susceptibility genes can be searched in the genome. Besides, due to many factors, such as regional diversity, ethnic differences, study sample size and gene interaction, inconsistent research conclusions is a commonplace, resulting in limited clinical reference value. Most studies have been conducted in a single population, so further analysis of the correlation between different populations is essential.

Currently, only a handful of NIHL susceptibility genes have been uncovered, and existing studies suggest that NIHL may be caused by accumulative abnormal influence of multiple genes. Further in-depth researches are needed to explore gene-gene interaction and find comprehensive and dominant susceptibility genes among numerous NIHL susceptibility genes. Although there are still great difficulties and challenges in the study of NIHL susceptibility genes, with the further research on new genetic research methods, such as next-generation DNA sequencing (NGS) and high-throughput genotyping array, more susceptibility genes related to NIHL will be found. The luminant prospect of designing of molecular probes that can be used for clinical detection of NIHL susceptible individuals is awaiting on the way.

In conclusion, genetic factor plays a vital role on the pathogenesis of NIHL. NIHL susceptible genes can be used for better identification of potential risks and prevent the occurrence of NIHL. Through the continuous screening of genetic variants in the susceptibility of NIHL, new susceptibility genes will come to light, and ideally, get into the stage of clinical application, which lays a solid foundation for the accurate screening of high-risk population and the reduction of NIHL incidence.

## Author Contributions

X-MC and X-MX drafted the manuscript. NY, W-WG, and S-LY collected the literature. X-MX summarized the literature. X-MC tabulated the data and drew the figures. Q-QJ revised the manuscript. Q-QJ and S-MY took responsibility for the integrity of the data and the accuracy of the manuscript. All authors contributed to the article and approved the submitted version.

## Conflict of Interest

The authors declare that the research was conducted in the absence of any commercial or financial relationships that could be construed as a potential conflict of interest.

## Publisher’s Note

All claims expressed in this article are solely those of the authors and do not necessarily represent those of their affiliated organizations, or those of the publisher, the editors and the reviewers. Any product that may be evaluated in this article, or claim that may be made by its manufacturer, is not guaranteed or endorsed by the publisher.
